# A Deep Learning Approach for the Classification of Fibroglandular Breast Density in Histology Images of Human Breast Tissue

**DOI:** 10.3390/cancers17030449

**Published:** 2025-01-28

**Authors:** Hanieh Heydarlou, Leigh J. Hodson, Mohsen Dorraki, Theresa E. Hickey, Wayne D. Tilley, Eric Smith, Wendy V. Ingman, Ali Farajpour

**Affiliations:** 1 Discipline of Surgical Specialties, Adelaide Medical School, University of Adelaide, The Queen Elizabeth Hospital, Woodville South, SA 5011, Australia; hanieh.heydarlou@adelaide.edu.au (H.H.); leigh.hodson@adelaide.edu.au (L.J.H.); wendy.ingman@adelaide.edu.au (W.V.I.); 2 Robinson Research Institute, University of Adelaide, Adelaide, SA 5006, Australia; 3 School of Computer and Mathematical Sciences, The University of Adelaide, Adelaide, SA 5005, Australia; mohsen.dorraki@adelaide.edu.au; 4 Australian Institute for Machine Learning (AIML), Adelaide, SA 5000, Australia; 5 Dame Roma Mitchell Cancer Research Laboratories, Adelaide Medical School, University of Adelaide, Adelaide, SA 5005, Australia; theresa.hickey@adelaide.edu.au (T.E.H.); wayne.tilley@adelaide.edu.au (W.D.T.); 6 Medical Oncology, Basil Hetzel Institute, The Queen Elizabeth Hospital, Woodville South, SA 5011, Australia; eric.smith@adelaide.edu.au

**Keywords:** convolutional neural networks, vision transformers, breast density, deep learning, hematoxylin and eosin (H&E)-stained breast tissue, medical image classification

## Abstract

Background: To progress research into the biological mechanisms that link mammographic breast density to breast cancer risk, fibroglandular breast density can be used as a surrogate measure. This study aimed to develop a computational tool to classify fibroglandular breast density in hematoxylin and eosin (H&E)-stained breast tissue sections using deep learning approaches that would assist future mammographic density research. Methods: Four different architectural configurations of transferred MobileNet-v2 convolutional neural networks (CNNs) and four different models of vision transformers were developed and trained on a database of H&E-stained normal human breast tissue sections (965 tissue blocks from 93 patients) that had been manually classified into one of five fibroglandular density classes, with class 1 being very low fibroglandular density and class 5 being very high fibroglandular density. Results: The MobileNet-Arc 1 and ViT model 1 achieved the highest overall F1 scores of 0.93 and 0.94, respectively. Both models exhibited the lowest false positive rate and highest true positive rate in class 5, while the most challenging classification was class 3, where images from classes 2 and 4 were mistakenly classified as class 3. The area under the curves (AUCs) for all classes were higher than 0.98. Conclusions: Both the ViT and MobileNet models showed promising performance in the accurate classification of H&E-stained tissue sections across all five fibroglandular density classes, providing a rapid and easy-to-use computational tool for breast density analysis.

## Simple Summary

Mammographic breast density is an important risk factor for breast cancer. Women with dense breasts have a high abundance of fibroglandular breast tissue, which can be seen on a mammogram and is associated with a greater risk of developing breast cancer. To progress research into the biological mechanisms that link mammographic density to breast cancer risk, fibroglandular density can be used as a surrogate measure. Fibroglandular density can be evaluated using thin formalin-fixed paraffin-embedded breast tissue sections stained with hematoxylin and eosin. To date, the classification of fibroglandular breast density is not automated and relies on visual assessment by researchers. Hence, this study explored the use of deep learning models to automate the classification of fibroglandular breast density.

## 1. Introduction

Mammographic breast density refers to the visual appearance of the breast on an X-ray mammogram and relates to the relative amount of fibroglandular versus adipose tissue [1]. Breast tissue with a high relative abundance of fibroglandular tissue shows up white on mammograms, while breast tissue with a high relative abundance of adipose tissue appears dark. Breast tissue that appears mostly white on a mammogram is considered extremely dense, and breast tissue that appears mostly dark is considered non-dense or mostly fatty. Mammographic breast density is a strong independent risk factor for breast cancer; compared with females with mostly fatty breasts, females who have extremely dense breasts have a 4- to 6-fold elevated risk of breast cancer when body mass index and age are matched [2,3]. Understanding the biological mechanisms that regulate mammographic breast density and breast cancer risk has the potential to provide new therapeutic opportunities for breast cancer prevention [4].

Mammographic breast density is a radiological finding extracted from a 2-dimensional mammogram image. It refers to the overall whiteness of the image, although there can be significant heterogeneity in mammographic density within an individual breast [5]. Mammographic breast density can be classified through the Breast Imaging Reporting and Data System (BIRADS), which defines four categories through visual and subjective classification by the radiologist [6]. There are also established quantitative methods for classifying mammographic breast density based on the integration of texture, gray-level information [7], and image digitalization. Both current qualitative and quantitative approaches, however, come with limitations. Earlier is less reproducible and the latest exaggerates the extent of density [2]. Deep learning techniques have been successfully applied to classify mammographic breast density on mammograms [8] and ultrasound images [9].

Research on the underlying biology of mammographic breast density is hampered by the difficulty of defining the mammographic density of small tissue samples, which may not represent the density of the whole breast. To overcome this limitation, fibroglandular breast density has been used for research purposes as a surrogate measure for mammographic density [4]. Unlike mammograms, which are used as a non-invasive technique for breast cancer diagnosis and breast density analysis, H&E-stained sections are derived from tissue samples taken during biopsy or surgery, making them invaluable sources for more detailed analysis [10]. H&E-stained classified sections enable researchers to investigate the biological mechanisms underlying mammographic breast density by comparing AI-classified high- and low-density breast tissues. Discovering novel biomarkers and pathways involved in mammographic breast density leads to novel therapeutic and prevention approaches for breast cancer.

To the best of our knowledge, no machine learning model has been developed yet for classifying fibroglandular breast density in H&E stained sections [11]. Convolutional neural networks (CNNs) have become the leading tools for classification tasks in computer-aided diagnostic systems for medical applications [12]. Particularly, CNN-based approaches have been successfully employed for extracting characteristic features from histopathology images of breast parenchymal tissue [10]. Previously, CNN-based models have been used to classify H&E-stained breast tissue samples based on tumor type [11]. MobileNet-v2, a specific CNN architecture, has demonstrated promising outcomes in medical image classification [13]. MobileNet-v2 is pre-trained on millions of images from the ImageNet dataset, enabling it to perform effectively even with limited data to other currently available CNN architectures [14]. In contrast, vision transformers [15] are a modern edition of neural networks that utilize a self-attention mechanism originally developed for natural language processing tasks but later showed their potential capability for image classification [16], particularly for histopathology, ultrasound, and mammography images [17]. ViTs are proving to be a valuable tool for a broad range of tasks including classification, object detection, and image segmentation [18].

In this paper, deep learning algorithms have been developed for the classification of fibroglandular breast density in H&E-stained formalin-fixed paraffin-embedded (FFPE) sections of human breast tissue using a transferred and modified version of MobileNet-v2 and a ViT model. FFPE refers to a tissue preparation technique in which human samples are fixed in formalin and embedded in paraffin for preservation and detailed microscopic analysis, respectively. Using a standard deep learning algorithm to classify H&E-stained sections by avoiding subjective errors and providing a consistent approach would enhance the robustness of data generated in this field.

## 2. Materials and Methods

This study received ethics approval from the Central Adelaide Local Health Network Human Ethics Research Committee (TQEH Ethics Approval #2011120) and the University of Adelaide Human Ethics Committee (#H-2014-175).

### 2.1. Tissue Processing

Women aged between 18 and 75 attending The Queen Elizabeth Hospital (TQEH) for prophylactic mastectomy or reduction mammoplasty were consented for the study. The tissue was confirmed as healthy non-neoplastic by the TQEH pathology department. The validation sample set was collected following informed consent from women undergoing breast reduction surgery at the Flinders Medical Centre, Adelaide, SA. Breast tissue was dissected into small pieces using surgical scalpel blades. Breast tissue then was fixed in 4% paraformaldehyde (Sigma-Aldrich; 3050 Spruce Street, St. Louis, MO 63103, USA, Cat# P6148), for 7 days at 4 °C, washed twice in PBS (1X), and transferred to 70% ethanol until further processing. Tissue was processed using the Excelsior tissue processor (Thermo Fisher Scientific;168 Third Avenue, Waltham, MA 02451, USA) followed by the dehydration, clearing, and embedding protocol: incubation in 70%, 80%, and 90% ethanol for an hour each, proceeded with incubation in 100% ethanol with 3 changes, 1 h each, and xylene with 3 changes, 1 h each. Finally, tissue was filtrated in paraffin wax with 3 changes, 1 h each. The resulting formalin-fixed paraffin-embedded (FFPE) tissue blocks were stored at room temperature before sectioning.

### 2.2. Hematoxylin and Eosin (H&E) Staining

Five-micrometer sections were cut from FFPE blocks using a microtome (Leica Biosystems; 495 Blackburn Road, Mount Waverley, VIC, Australia). These sections were then floated onto a warm (42 °C) water bath and transferred to super adhesive glass slides (Trajan Series 3 Adhesive microscope slides, Ringwood, Victoria, Cat#473042491). The slides were incubated at 37 °C overnight until fully dry. Sections were dewaxed through three changes in xylene (Merck Millipore, Frankfurter Str. 250, Darmstadt, Germany; Cat# 108298) and rehydrated through a gradient of 100%, 95%, 70%, and 50% ethanol, followed by distilled water. Tissue sections were stained with hematoxylin (Sigma Aldrich, St. Louis, MO, USA; Cat#HHS16) for 30 s and eosin (Sigma Aldrich, St. Louis, MO, USA; Cat#318906) for 5 s. Slides were then dehydrated with 100% and 95% ethanol and cleared with two changes in xylene. The tissue slides were then mounted using a mounting medium (Proscitech; 6/118 Wecker Road, Morningside, QLD, Australia; Cat#IM022). Finally, the stained slides were scanned using a digital Nanozoomer 2.0-HT slide scanner (Hamamatsu Photonics K.K. 325-6, Sunayama-cho, Higashi-ku, Hamamatsu-shi, Shizuoka, Japan, Adelaide, SA, Australia) with a 40X objective lens, generating high-resolution (0.23 µm) images for computer-based analysis.

### 2.3. Fibroglandular Breast Density Score Classification

**Figure 1:**
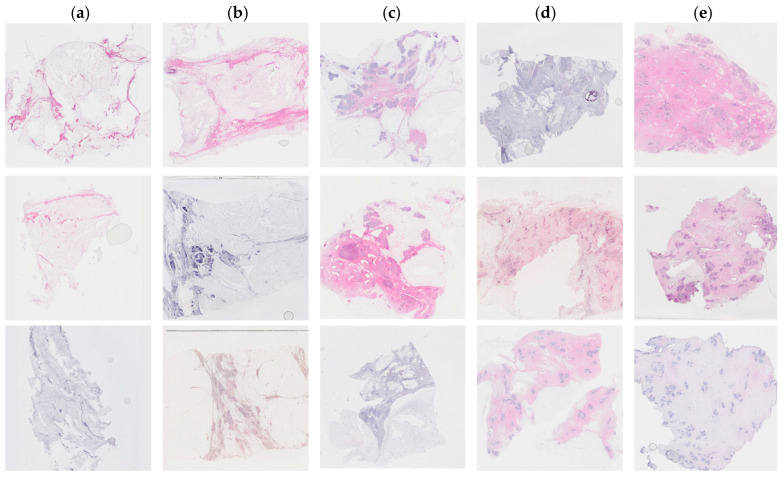
Examples of hematoxylin and eosin-stained breast tissue specimens across five density classes. (**a**) Breast tissue with 0–10% fibroglandular tissue, representing class 1. (**b**) Breast tissue with 11–25% fibroglandular tissue, representing class 2. (**c**) Breast tissue with 26–50% fibroglandular tissue, representing class 3. (**d**) Breast tissue with 51–75% fibroglandular tissue, representing class 4. (**e**) Breast tissue with 76–100% fibroglandular tissue, representing class 5.

Tissue staining was performed by multiple laboratory specialists over several years, which may result in variations in staining intensity across the images (Figure 1). The staining protocol and reagents used remained consistent throughout this study. These images were used to set up the training and test database.

A validation study was performed using H&E-stained human breast tissue independently collected from a different laboratory that was not part of the training and test dataset. These de-identified H&E-stained breast tissue sections are described in the validation section of the Results.

Each patient had an average of 10 tissue blocks. One tissue section was assessed in each FFPE tissue block. In total, 965 images were collected from 93 patients. A panel of scientists (HH, LH, WI) classified each image semi-quantitatively. The panel reached a consensus on density through discussion. Higher density scores were assigned to sections containing a greater percentage of stroma and epithelium and a smaller amount of adipose tissue. The fibroglandular density classification scale was defined by Archer [5] and demonstrated a correlation with mammographic breast density in tissue samples obtained by X-ray image-guided biopsy [19]. The classification scale assigned each tissue sample to a number between 1 to 5, where 1 represented 0–10%, 2 represented 10–25%, 3 represented 25–50%, 4 represented 50–75%, and 5 represented >75% of fibroglandular tissue (Figure 1).

### 2.4. Image Pre-Processing

A total of 965 high-resolution original images were generated from the human breast tissues. All H&E-stained images were resized to 224 × 224 pixels. Then, for processing and manipulation, the images were converted into an array format and stored in a data frame. Libraries including sci-kit-learn 0.23.2, Pandas 1.5.3, and Numpy 1.23.5 were used for pre-processing the images. Diverse data augmentation techniques were applied to improve model generalization and expand the training dataset [20]. These techniques included horizontal flipping, vertical flipping, and rotating each image by 0, 90, 180, and 270 degrees. As this work focuses on the breast density classification of tissue sections and not on the density classification of the whole breast, the choice of rotation angles for data augmentation is not restricted and can be extended beyond small magnitudes. However, for mammography density classification of mammograms, it is recommended to limit the rotation angle to 0–15 degrees as higher rotations might alter the biological relevance and spatial relationships between the fundamental tissue structures. It should be noted that cropping an H&E-stained image changes its breast density score, and thus if cropping is used as an augmentation method, it requires a fibroglandular breast density re-assessment.

**Figure 2:**
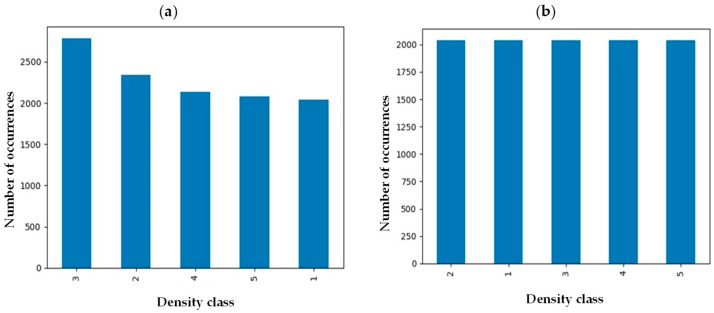
Breast density distribution (**a**) before and (**b**) after undersampling balancing.

Medical images from many individuals come with intrinsic imbalance, where some classes of fibroglandular density will be represented in the sample set more than others. To ensure each class represents itself properly, images in each class were evened out by implementing an undersampling strategy to reach an equal data distribution (Figure 2) [21]. Before implementation of the balancing technique, the number of samples in class 3 was maximum while density classes 1 and 5 were the minority classes. However, the number of samples in each class was around 2000 after undersampling balancing, preventing potential bias toward a specific class.

### 2.5. Deep Learning Model

We introduce a unique data augmentation approach specifically tailored to images of H&E-stained human breast tissue sections, offering significant benefits over traditional techniques used for mammography images. Unlike mammograms, where data augmentation is often restricted to small rotation angles (0–15 degrees) to preserve diagnostic relevance and the natural structure of the whole breast, our method allows for rotations at any angle. Meanwhile, cropping is not suitable for human tissue sections, as it can change the composition of fibroglandular and adipose tissues, potentially altering the breast density score. To address variability in H&E-stained images arising from differences in laboratory protocols, we applied stain normalization as part of our analysis. In addition to data augmentation, no ViT model has yet been developed for the breast density classification of H&E-stained human breast sections. Moreover, our modification of the MobileNet-v2 model is both unique and specific to H&E images of human breast tissues. Eight different layers are added to the existing MobleNet-v2 model to specifically tailor it for the breast density classification in histopathology images. All the existing conventional deep-learning models of breast density classification are limited to mammograms and are not applicable to H&E-stained tissue sections.

#### 2.5.1. MobileNet-v2

This study implemented a convolutional neural network (CNN), backend Tensorflow (version 2.12.0), using the sequential Keras library, with MobileNet-v2 architecture. MobileNet-v2 is a pre-trained CNN with 53 different layers in depth [14] and has been trained on over a million images from the ImageNet database [22]. The model was developed and trained using TensorFlow 2.15.0.

In this application, the final fully connected layer of the MobileNet-v2 was excluded, allowing for adjustments based on the specific needs of our target application (breast density classification). Meanwhile, the initial layers of the model were fixed during training by freezing their parameters.

**Figure 3:**
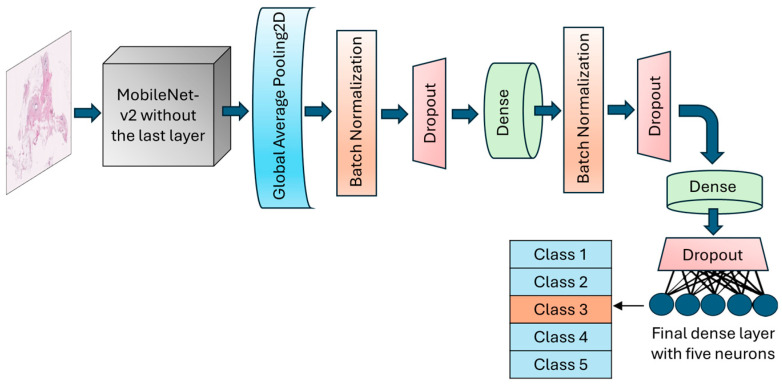
Schematic of the architecture of the MobileNet-v2 model. The top layer of the transferred model was removed, and a number of various layers were added to allow for the trainability of the H&E-stained breast tissue sample.

The GlobalAveragePooling2D layer was called to reduce the dimensionality and number of parameters by computing the average from each feature map to a single value. BatchNormalization layer and dropout layers with a rate of 30% were added after the global average pooling layer to normalize batches and prevent potential overfitting, respectively. An intermediate dense layer with 64 neurons and ReLU activation was added to account for nonlinearity and handle complex patterns. The final dense layer had 5 neurons with softmax activation to conduct the muti-class classification and calculate the probability of each fibroglandular breast density class (Figure 3). The learning rate was designed to change based on an exponential decay, beginning with an initial value of 0.001 and followed by a decay rate of 0.9. To optimize model weights, the model uses an Adam optimizer with a loss function setting to categorical cross-entropy. An early stopping callback was used to terminate the training process when validation losses did not decrease for 10 epochs in a row. However, the training could continue for a maximum of 200 consecutive epochs if necessary.

#### 2.5.2. Vision Transformer

Vision Transformer [15] is an emerging type of deep neural network model based on transformer encoders with a self-attention mechanism [15]. ViT showed stronger capabilities compared to the previous model using sequences of image patches to classify the full image [16]. ViTs work by dividing an image into small fixed-size patches, which are linearly embedded and fed into a number of transformer encoders to extract image features.

**Figure 4:**
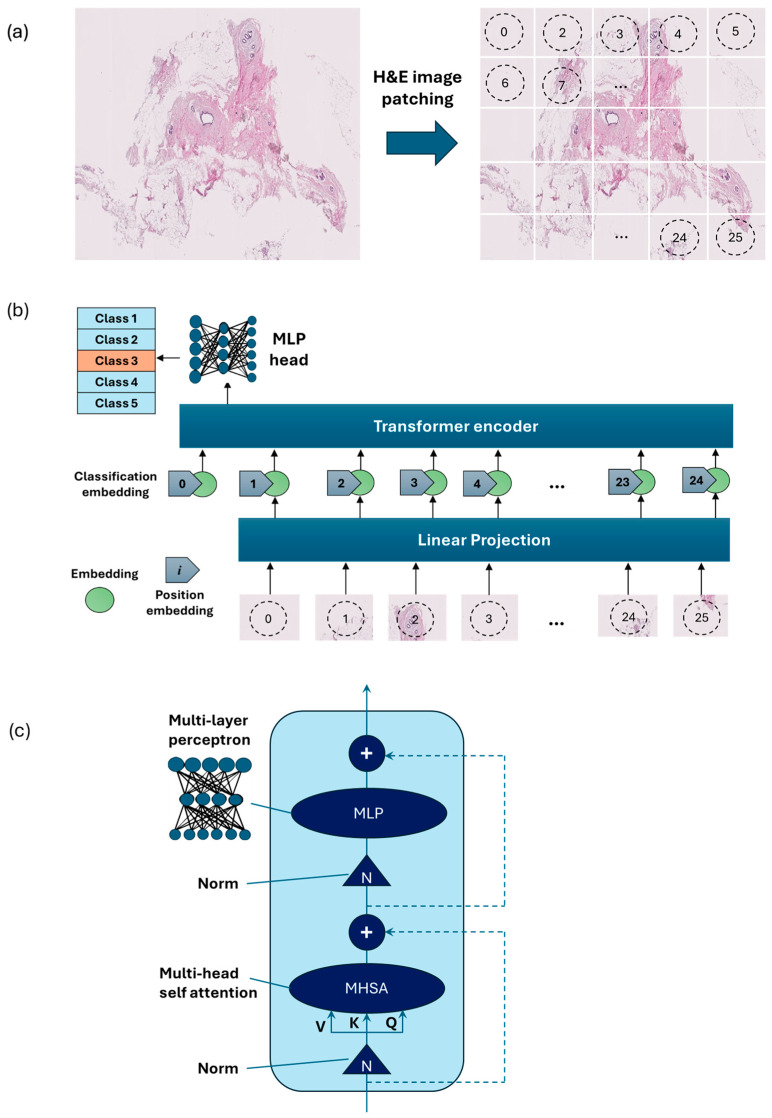
Overview of the architecture of the vision transformer model: (**a**) H&E-stained image patching, (**b**) linear projection, embedding, and position embedding, followed by processing within the transformer encoder and MLP head, (**c**) schematic of a transformer encoder consisting of a normalization layer, multi-head self-attention (MHSA), and MLP.

As an alternative to the MobileNet-v2 model, a ViT model was developed using PyTorch version 2.3.1 and CUDA version cu118 to allow for the use of a graphics processing unit (GPU) to accelerate the training process. As ViT models demand large training data, we applied larger image augmentation in our database including small adjustments to brightness, contrast, and saturation on images plus random erasing. Random erasing removed a small portion of an image with a possibility of 50%, ranging between 1% and 7% of the image, and an aspect ratio of 0.3 to 3.3 to resemble human technical errors. In this study, we set the patch size to 16, model depth to 12, and attention head to 8. Patch and position embedding were implemented with an internal feature dimension of 64. The multi-layer perceptron (MLP) part had a hidden layer size of 64 pixels, and a dropout rate of 0.1 was applied to both the overall and embedding dropouts. An overview of the ViT model used for the classification of H&E-stained images of human breast tissue is shown in Figure 4. For the loss function, we applied the cross-entropy and to optimize the model, and an Adam optimizer with a learning rate of 0.001 was applied. Moreover, we applied a learning rate scheduler, using the PyTorch StepLR scheduler, decaying the learning rate by a factor of gamma equal to 0.7. In this model, patience was considered 15, which stops the training process when validation losses do not decrease after 15 epochs.

## 3. Results

### 3.1. MobileNet-v2 Evaluation

The MobileNet-v2 model was transferred without the top layer. The input shape was set to (224, 224, 3). The weights of the transferred model were taken as those of the pre-trained model [22]. One two-dimensional global average pooling layer, batch normalization layer, and dropout layer (rate = 0.3) were added, followed by a fully-connected layer (ReLU activation, neurons = 128). Batch normalization and a dropout layer of 0.4 were used after the dense layer to avoid overfitting. Another dense layer with 64 units and ReLU activation, followed by a dropout layer of 0.3 rate was implemented. The output layer was a fully connected layer with 5 neurons (the number of breast density classes). As multi-class classification was conducted, the softmax activation was used in the last layer. The base layers of the transferred MobileNet-v2 model were frozen, keeping its parameters untrainable.

Table 1 shows the summary of hyperparameters applied for the MobileNetV2. The batch size was set to 32. The learning rate adjusted to 0.001, with a step size of 1 reduced by a decay rate of 0.9. The patience was designed to 10. Training would have continued to a maximum of 200 epochs, although it could stop after 10 epochs if the validation loss was not improved. The categorical cross-entropy loss function, exponential scheduler, and Adam optimizer were used in the training process.

We evaluated the architectures of four different transferred MobileNet-v2 models (MobileNet-Arc 1, 2, 3, and 4; Table 2). The top layer of the MobileNet-v2 was removed, and a number of various layers were added, allowing for the transferred model to learn from the training H&E dataset. One GlobalAveragePooling2D layer was added for all architecture models of MobileNet-V2. MobileNet-Arc 2 and 3 were almost similar in the number of used layers, but MobileNet-Arc 3 was the only architectural model using Cov2D and MaxPooling2D layers. MobileNet-Arc 4 had a higher number of added dense layers and dropout layers compared to others. MobileNet-Arc 3 with more than 3,000,000 total parameters was the most complex model with many internal configurations. Other architectural models held around 2,300,000 to 2,400,000 total parameters. MobileNet-Arc 1 with three added dense and dropout layers in addition to 2 BatchNorm layers contains more total layers compared to MobileNet-Arc 2 and 3 and less than MobileNet-Arc 4, using the medium number of trainable parameters. MobileNet-Arc 2 had the smallest number of trainable parameters, which came from having fewer layers (Table 2).

Different metrics were used to monitor and measure the performance of a model during training and testing. These parameters helped us to describe how well the model generalizes. The most important metrics for the performance analysis of classification tasks were accuracy and loss. Accuracy is defined as the ratio of all correct predictions to all predictions. Loss indicates the quantification of errors between the model’s results and true positives (TPs). The training dynamics were monitored by tracking the accuracy and loss metrics throughout the training process.

**Figure 5:**
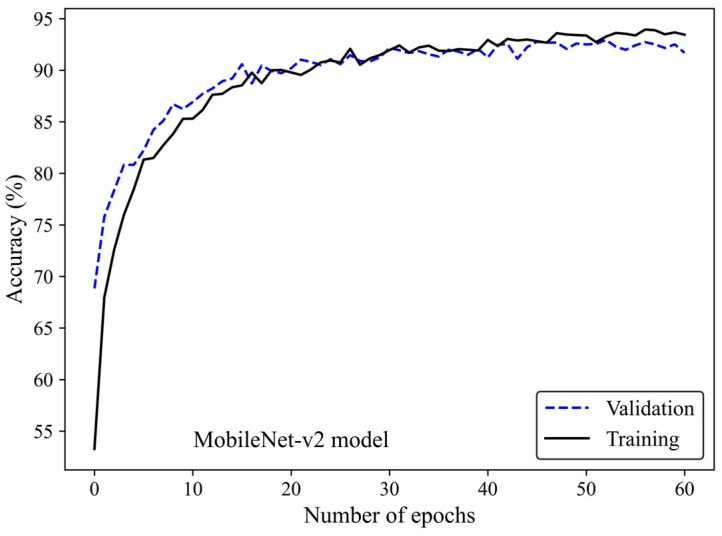
Accuracy score versus epoch for the training and validation data using MobileNet-Arc 1 configuration.

Figure 5 shows the accuracy curve of the MobileNet-v2 model. In early epochs, the validation accuracy was greater than the training accuracy. As the number of epochs increased, the training and validation accuracies improved. The difference between the validation and training accuracy decreased by training the model on a higher number of epochs. The small gap between the validation and training accuracies indicated that the model effectively prevented overfitting.

**Figure 6:**
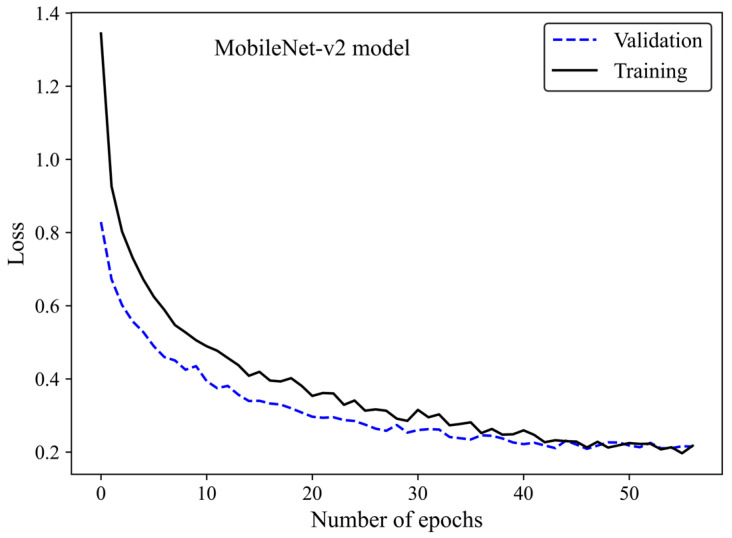
Loss versus epochs for the training and validation data using the MobileNet-Arc 1 configuration.

The loss curve (Figure 6) demonstrates the MobileNet-v2 model’s learning process by increasing the number of epochs. The x-axis represents the number of epochs, whereas the y-axis is the loss. The model gradually learns from the training H&E-stained image dataset as evidenced by the reduction in the training loss with increasing epochs. The validation loss is an indicator of the model’s performance on unseen H&E-stained breast images. It is found that both the training and validation loss decreased by training the modified transferred model using a greater number of epochs. No sign of overfitting was observed. Validation loss decreased along with training loss, indicating the generalization capability of the model. The simultaneous reduction in the validation and training loss shows that the model performs well not only on the training images but also on new unseen H&E-stained images. This implies that the machine learning model does not just memorize the training data but has learned the essential patterns and features required for reliable generalization and accurate breast density classification of new images.

**Figure 7:**
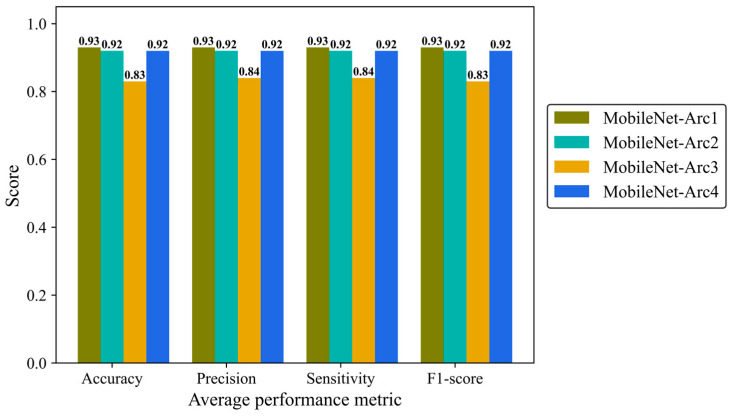
Accuracy, average precision, mean sensitivity (recall), and average F-1 score of the MobileNet-v2 model with four different architectures on the test H&E-stained images.

Precision, Sensitivity (recall), and F1-score were reported to give more details of the model performance. Precision is defined as a ratio of true positives to the total positive predictions including true (TP) and false positives (FP). Sensitivity (recall) is defined as the number of TP predictions divided by the total number of TP and false negative (FN) classifications. F1-score is the combination of precision and recall provides us with an overall overview of the model performance. We analyzed average metrics to make a comparison between different architectural configurations of the MobileNetV2 model. MobileNet-Arc 1 displayed the strongest overall classification performance, while MobileNet-Arc 3 showed the weakest. MobileNet-Arc 1 achieved an F1-score of 0.93, suggesting the model performs well on both precision and recall for positive cases. MobileNet-Arc 1 predicted the correct incidence for 93% of H&E-stained human breast images, introducing it as a reliable option for breast density classification. MobileNet-Arc 2 and 4 also represented a high-performing model with an F1-score of 0.92; however, MobileNet-Arc 3 with a score of 0.83 suggests that it may not be a very strong model (Figure 7).

We then evaluated the performance of each architectural model in classifying mammographic breast density into five classes using human breast histopathology samples (Table 3). All architectural models performed best in classifying class 1 and 5 fibroglandular breast density. In contrast, performance was lowest for class 3 across all models. MobileNet-Arc1 emerged as the top performer, achieving an F1-score of 0.96 for class 5 samples. MobileNet-Arc2 and 4 also produced satisfactory results, whereas MobileNet-Arc3 was found to have an unreliable architecture. MobileNet-Arc3 achieved the precision and sensitivity of 0.73 for class 3 and 4 samples, respectively. In MobileNet-Arc3, the F1-score for all categories recorded less than 0.90, and class 3 had the lowest F1-score (0.78) among all classes in all architectures (Table 3).

**Figure 8:**
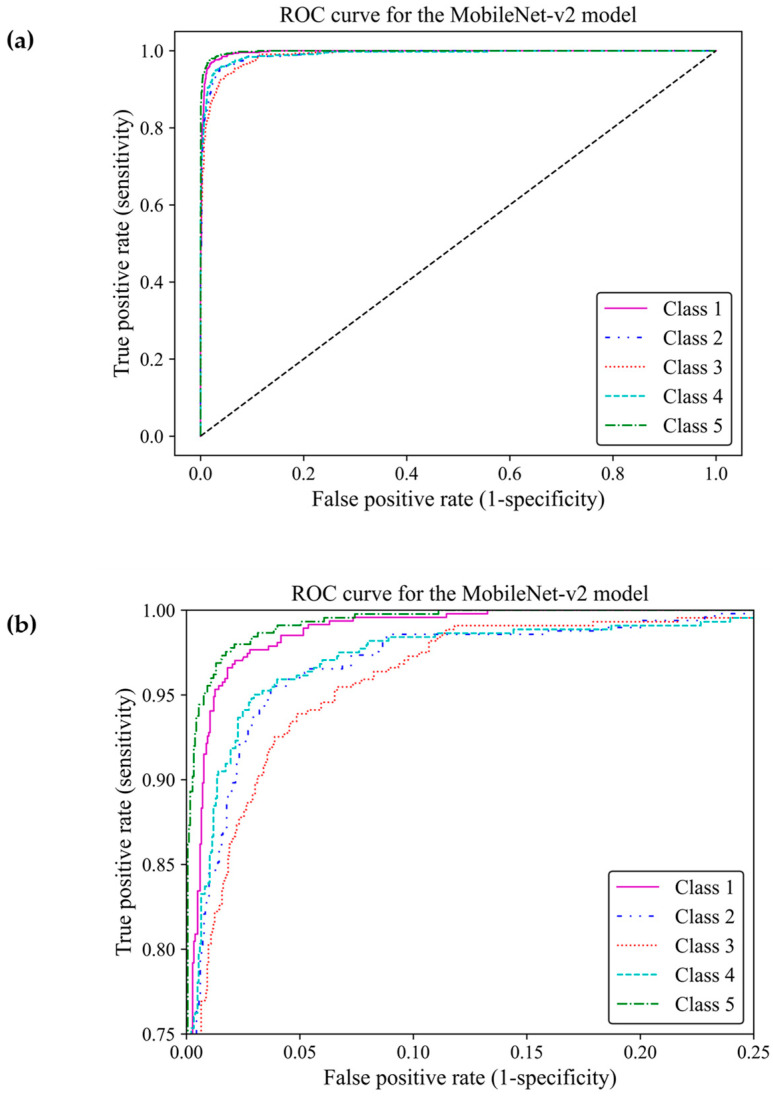
Receiver operating characteristic curve of the transferred and modified MobileNet-Arc 1 model for the five different breast density classes. (**a**,**b**) A zoomed-in overview of the ROC curve, focusing on the TPR ranging from 0.75 to 1.

When classifying medical data, it is vital to understand the number of true positive predictions for each class as the cost of false positives. Here, the receiver operating characteristic curve [23] and confusion matrix were used to evaluate the effectiveness of the model to distinguish between the breast density classes by showing actual true predictions and errors in each class. The ROC curve illustrates the trade-off between true positive rates (TPR) and false positive rates (FPR) for various thresholds. TPR indicates a ratio of actual positive predictions that are correctly identified by the model as positive, and FPR represents the ratio of actual negative predictions that are incorrectly identified by the model as positives. TPR and FPR range from 0 to 1. The area under the curve (AUC) of classes 1, 2, 3, 4, and 5 were estimated as 0.996, 0.989, 0.990, 0.989, and 0.997, respectively, all of which were close to one. This demonstrated that the MobileNet-Arc1 model performed well in effectively distinguishing between breast density classes. The ROC curves of all classes achieved a high TPR and a low FPR as shown in Figure 8a, indicating that the transferred MobileNet-v2 Arc 1 model was capable of classifying the H&E-stained histopathology human breast sample into the five different breast density classes. The model performed best in class 5, closely followed by class 1. The model showed the weakest performance in class 3 but it is still considered a well-designed classifier (Figure 8b).

**Figure 9:**
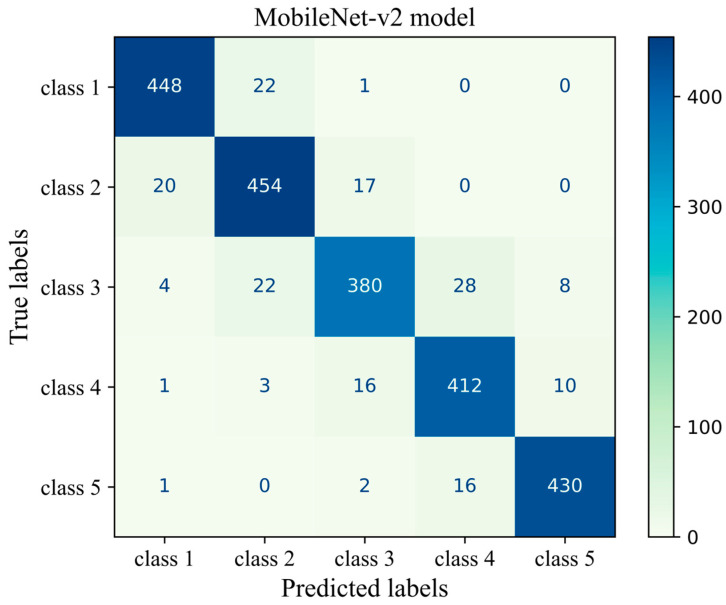
Confusion matrix of the MobileNet-v2 model (MobileNet-Arc 1) on the test dataset.

Figure 9 shows the confusion matrix of the transferred CNN model on the unseen H&E-stained images (test dataset). This heatmap provides details about the performance of the deep learning model across different mammographic density classes. The sum of each row indicates the number of true labels for a given density class, while the total number of predicted labels is the sum along the column direction. Each cell indicates the number of H&E-stained images with their true (row name) and predicted (column name) labels. Diagonal cells are the number of histopathology images that were accurately predicted by the MobileNet-v2 model for each density class while an off-diagonal cell indicates the number of H&E-stained images that were incorrectly classified by the trained model. Generally, the breast density class for the majority of H&E-stained images was correctly predicted by the model. This was well evidenced by the high values along the diagonal cells and low values across the off-diagonal cells within the confusion matrix. The most correct prediction belongs to class 1 with 448 incidences out of 471 incidences. Among the misclassified slides, 22 were incorrectly identified as class 2 and 1 was misclassified as class 3. No slides were misclassified as classes 4 or 5. The weakest performance is related to class 3 where the model correctly classified an incidence of 380 from the total incidence of 442 and misclassified 62 incidences, most of which were misclassified as classes 2 and 4.

### 3.2. Vision Transformer [15] Evaluation

Here, we present a comparative analysis of the performance of Vision Transformer [15] models across four different structural configurations on the five density classes.

An initial learning rate of 0.001 was used that gradually reduced over time with a decay factor of 0.7 and updated weights at every step (step size = 1). The training was limited to a maximum of 75 epochs, and it could be terminated if no progress was observed for 15 consecutive epochs. The Cross-Entropy Loss, Adam optimizer, and StepLR learning rate scheduler were employed for this ViT model (Table 4).

Four different ViT models were designed for this study. All models except model 2 were set to a patch size of 16 and an embedding dimension of 64. For the ViT model 2, both patch size and embedding dimension were set to 32. The ViT model 4 minimized the number of trainable parameters by using a depth of 3, attention heads of 2, and an MLP dimension of 16 in the transformer encoder. The ViT model 1 had the largest trainable parameters and a complexity level of 64 within its MLP. The overall dropout and embedding dropout rates were set to 0.1 for the ViT models 1 and 2, and 0.05 and 0.02 for the ViT models 3 and 4, respectively (Table 5).

**Figure 10:**
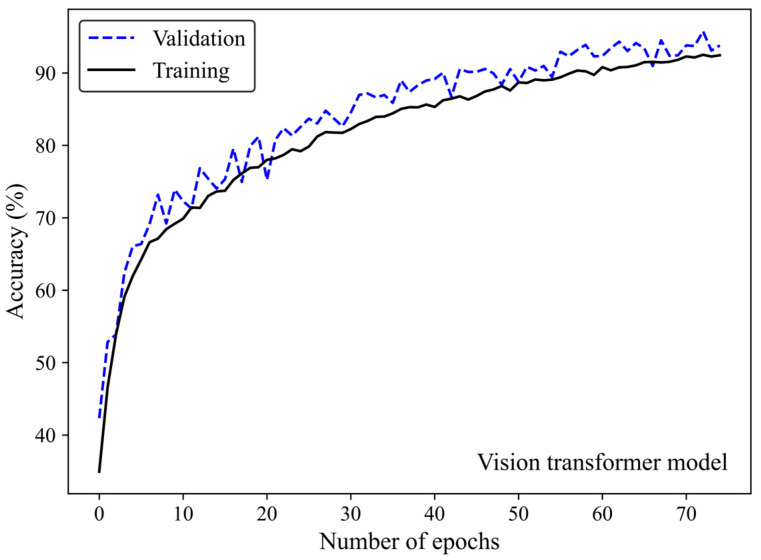
Accuracy score versus epoch for the training and validation data using the ViT model 3.

**Figure 11:**
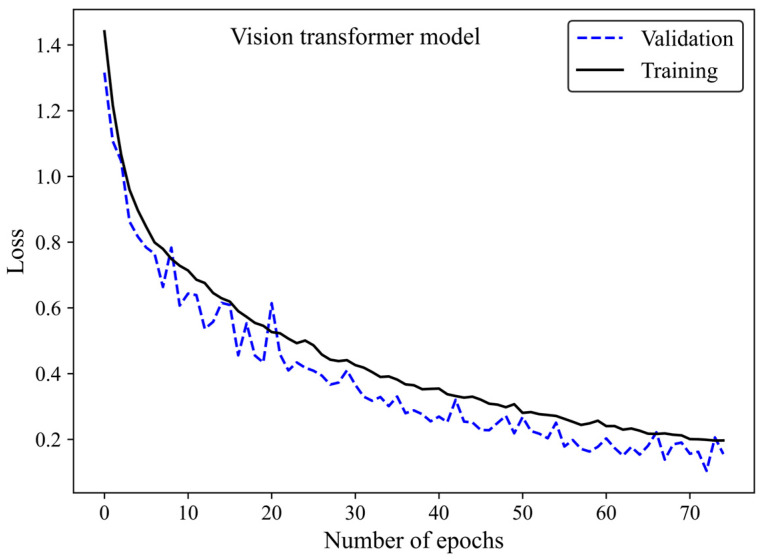
Loss versus epoch for the training and validation images using the ViT model 3.

Accuracy and loss were monitored to see how the ViT model learns and adapts over time. Along with increasing the number of epochs, the number of accurate predictions improved (Figure 10) whereas the model’s loss decreased (Figure 11), highlighting the efficiency of the learning process.

**Figure 12:**
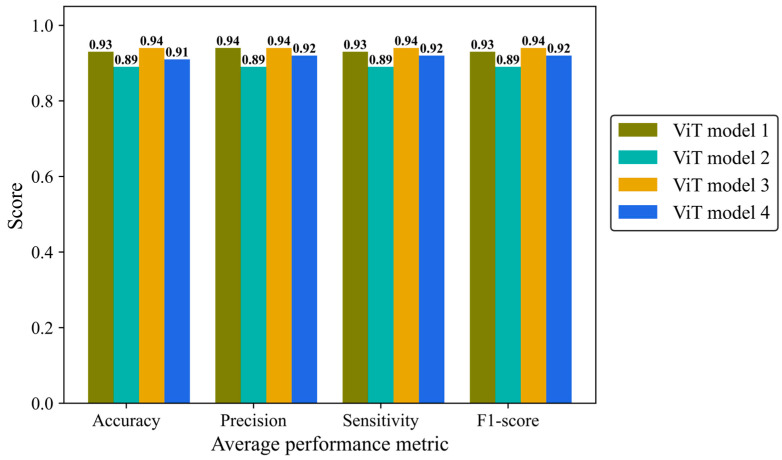
Accuracy, average precision, mean sensitivity (recall), and average F-1 score of the vision transformer model with four different architectures on the test H&E-stained images.

Accuracy, average precision, sensitivity (recall), and F1-score were monitored separately in the four architectures of the ViT model (Figure 12). ViT model 3 is the most effective architecture, achieving a score of 0.94 in all mentioned metrics followed closely by the ViT model 1 and ViT model 4 with average scores of 0.93 and 0.92, respectively. ViT model 2 did not yield the desired result, with a score of 0.89 in all metrics.

Table 6 shows the precision, sensitivity, and F1-score for each of the breast density classes, for all ViT models. All models had their best performance in class 5. Model 1 stood out with a remarkable sensitivity of 0.99 in class 5 while the ViT models 2 and 3 had the lowest sensitivity in class 5 (0.91). The F1-score ranged from 0.87 to 0.97. The lowest F1-score was related to class 2 in all models. In alignment with the other results, model ViT 2 achieved three F1-scores below 0.90 linked to classes 2, 3, and 4, exhibiting the least satisfactory performance among different ViT architectures.

The ViT model was designed for a multi-class classification. The average metrics do not represent each class’s performance. To evaluate how well this model distinguishes density between different classes, we depicted the confusion matrix and ROC curve.

**Figure 13:**
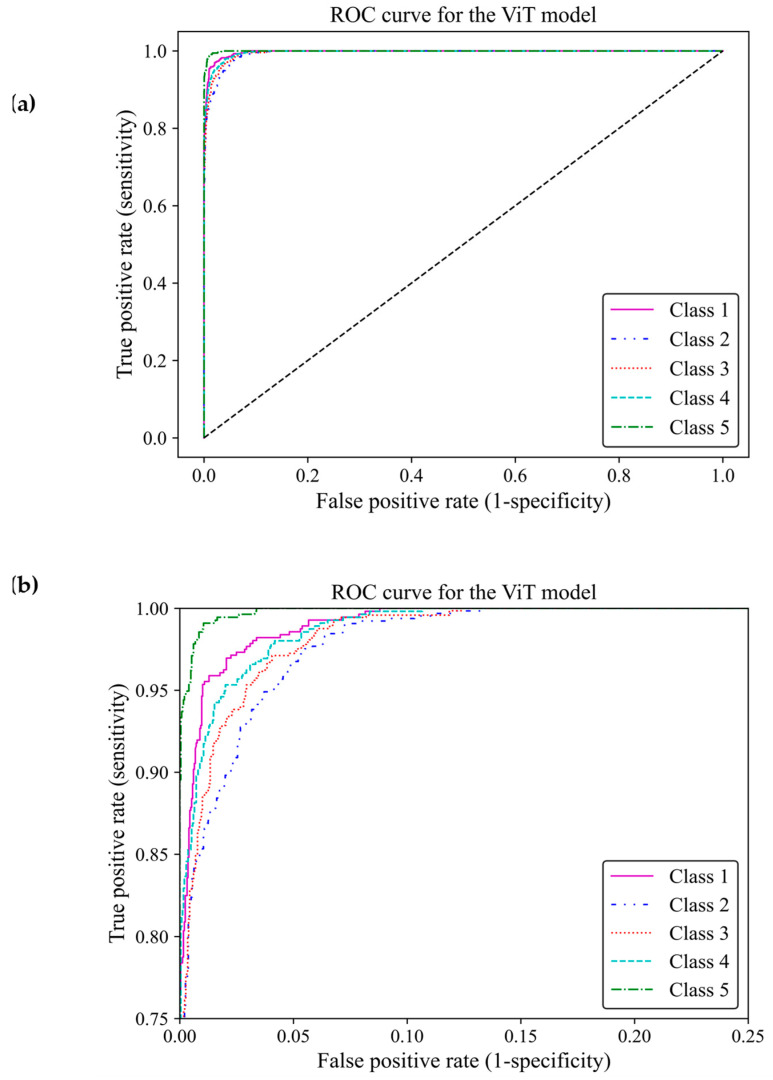
ROC curve of the vision transformer model for the five different fibroglandular breast density classes. (**a**) The ROC curve indicates the TPR within the range of 0 and 1 versus the FPR. (**b**) A zoomed-in overview of the ROC curve, focusing on the TPR ranging from 0.75 to 1. This chart provides more details on the performance of the ViT model 3 at higher TPRs.

Based on ROC classification, all classes achieved desirable results (Figure 13a). In the zoom-in figure, we can observe model classified cases with the lowest false positive and highest true positive predictions in class 5, which makes the model performance in this class almost perfect. The curve for class 1 displayed the nearest match to the class 5 curve, followed by curves of classes 4 and 3 exhibiting high similarity. Class 2 illustrated the least separation from a random classifier compared to other classes; however, it remained above chance levels for acceptable classification (Figure 13b). The area under the curve (AUC) of classes 1, 2, 3, 4 and 5 were 0.997, 0.989, 0.992, 0.993, and 0.998, respectively. All of these AUCs were close to one, indicating that the ViT model 3 had great capability to distinguish between different breast density classes.

**Figure 14:**
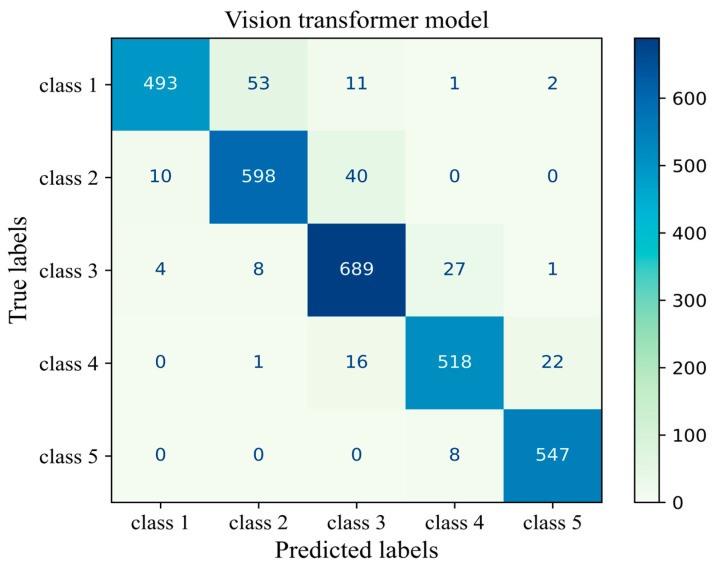
Confusion matrix of the Vit model 3 for the unseen test H&E-stained images of human breast tissue.

The number of true and false predictions in each class is presented (Figure 14). Model performance for class 5 is the strongest among all classes; it classified 547 cases of class 5 correctly and had just 8 wrong predictions, which belonged to class 4 and were misinterpreted in class 5. Classes 1, 2, 3, and 4 had 493, 598, 689, and 518 true positive predictions, respectively. All classes had the most false positive predictions with their neighboring class. For instance, the model had 40 wrong predictions belonging to class 3 but which were taken as other classes. The model had the weakest performance in class 1 with 67 wrong predictions, the majority of which wrongly predicted to belong to class 2 (Figure 14). The results of the validation of this study are given in Appendix A.

## 4. Discussion

Breast cancer is the most commonly diagnosed cancer in women and the incidence is rising across all age groups [24,25]. Current research that aims to reduce breast cancer risk and improve early detection is increasingly using data-intensive approaches, which rely on computational methods for analysis. Although this brings invaluable information, the generation of large amounts of data can make it difficult to analyze information. Deep learning, using artificial neural networks that simulate the human brain [26], has emerged as a breakthrough approach to support medical research including single-cell transcriptomics, DNA sequencing, protein interactions, drug development, and disease diagnosis [27,28].

Early models of deep learning used extracted features and fed them into the model. More recently, deep learning models have used pre-trained databases through a technique known as transfer learning [29]. Models that use transfer learning benefit from a wide range of features learned from massive datasets. This makes them ideal for applications with limited data, such as medical image analysis [30]. Medical image analysis for breast cancer research can benefit from this advancement in deep learning, which is preparing the way for using image analysis algorithms to detect and diagnose breast cancer. Of significance, deep learning approaches can reduce radiologist screening time by triaging the digital mammogram images most likely to require recall and further assessment [31] and identify those women most at risk of a future breast cancer diagnosis [32,33]. Promising results from histopathological classification of breast biopsies suggest that deep learning could also be employed for quality control in breast cancer detection [34,35].

The application of deep learning in medical image analysis is not limited to breast cancer detection. Mammographic breast density classification reached radiologist-level accuracy through this advancement. Despite using standardized protocols and advanced digital imaging methods, operator miscalculation, variation in the operator’s perception, and a heavy workload can still lead to inaccuracies in mammographic density assessment [25]. To minimize these errors, new automated methods of mammographic density measurement have been developed that use a computerized analysis algorithm that improves the consistency of results [8,23,36]. Here, we use deep learning to develop an automated histology image analysis tool to classify fibroglandular breast density in H&E-stained FFPE tissue sections. Whilst this tool is relatively simplistic in comparison to other medical research applications of deep learning, it has the potential to provide a foundation for future applications in research image analysis.

This study investigated a CNN model with four different architectures of MobileNet-v2 and different models of ViTs to classify fibroglandular breast density in H&E-stained human breast tissue samples. CNNs are mature enough to be the most common deep learning approach in medical vision classification. CNNs offer highly accurate automated feature extraction from various medical image sources, such as mammograms, X-rays, and histopathology images. CNNs can classify images into multiple categories, which is well-suited for this research [37]. Moreover, employing pre-trained models of CNNs including MobilNetV2 is advantageous when dealing with limited data like labeled medical images as it minimizes the required training. MobileNet-v2 was particularly chosen due to its efficiency in terms of computational cost and memory usage, making it suitable for resource-constrained environments. Its depth wise separable convolutions allow for a significant reduction in the number of parameters while maintaining competitive performance, which is crucial for real-time applications [14].

Vision Transformer (ViT), on the other hand, was selected for its superior ability to model long-range dependencies and capture global contextual information, which are essential for complex feature representations. ViT has demonstrated state-of-the-art performance in various vision tasks and offers advantages in handling diverse and complex data compared to traditional convolutional architectures [38]. Unlike CNNs, ViTs dynamically learn relationships across the entire image, enabling them to adapt to complex medical image classification tasks. The self-attention mechanism supports the model in focusing on the most relevant part of the image. ViTs are particularly well-suited for high-resolution large images with complex patterns [38]. Furthermore, ViTs can be integrated with other models, such as CNNs, to create more powerful hybrid architectures, highlighting the importance of further research into ViT-based models [39].

Both models achieved a high level of accuracy in classifying fibroglandular breast density (ViT model 3: 0.94 and MobileNet Arc 1: 0.93). However, their performance was not identical. MobileNet-v2 using convolutional layers achieved success across each of the evaluated architectural configurations, except MobileNet-Arc 3. The use of depthwise separable convolutions and residual linear bottlenecks substantially reduces the number of parameters and training time [40]. Additionally, it enables the model to learn effectively from smaller datasets and operate with lower computational resources, leading to deployment on mobile and embedded devices [40]. ViTs, using multi-head self-attention, can learn more characteristic features and deliver a high accuracy score. Four different models of ViT were evaluated, and almost all of them delivered a satisfactory result. The ViT approach for analyzing images is not just understanding individual image patches, but also the relationship between images without considering their distance within an image [41,42]. This allows ViT to generalize advanced models in image-analyzing tasks.

In agreement with other studies [43], we found that ViT excels in classifying medical images. Rather than improved general performance, ViT illustrates fewer incorrect predictions in classifying the fibroglandular breast density in each class. However, ViT required a larger dataset and a longer training process. As a result, ViT has a high computational cost for training caused by the intensive use of GPUs (Graphics Processing Units). Both models almost perfectly classified fibroglandular breast tissue samples in classes 1 and 5, while the most challenging classes are class 3 followed by classes 2 and 4. The challenge arises because sometimes, there is a narrow distinction between classes. Most errors arise when models classify data points that are close to the boundaries between neighboring classes. For instance, some images that truly belonged to class 3 were incorrectly classified as class 2 or 4, leading to increased prediction errors and diminished sensitivity for this class.

The limitations of this study are the relatively small sample size and the use of a private single-laboratory dataset. This limitation particularly impacts the performance of ViTs. We anticipate that with a larger and more diverse dataset, ViTs could achieve better overall performance, as they are inherently strong models. The lack of significant improvement in ViTs over MobileNet-v2 in this study is likely attributable to the constraints of the database. The dataset used in this study mainly consists of H&E-stained images from our laboratory. This limitation is particularly relevant in challenging classifications, such as class 3, where the model struggled to distinguish between classes due to insufficient diversity in the training data. To enhance robustness and distinguishing abilities, the models require using larger datasets from different laboratories.

## 5. Conclusions

This research has developed deep learning models for the classification of fibroglandular breast density, implementing MobileNet-v2 and vision transformers. The MobileNet-Arc 1 and model ViT 3 with accuracies of 0.93 and 0.94, respectively, were identified as the best architectural models. These results were validated by evaluating model performance on unseen H&E-stained sections prepared in another laboratory. The accuracy and F1-score of the deep learning models (both the ViT and MobileNet) slightly decreased from class 1 and 5 to intermediate classes such as class 3. This would highlight the inherent challenge in the precise definition of class 3, which might include a mix of overlapping characteristics. After performing a comprehensive analysis, we have found that ViT offers a slight performance improvement, although it requires a higher computational cost to achieve high accuracy, uses a larger number of parameters, and has a longer processing time. For large datasets where high accuracy is important, it is recommended to use ViT models to generalize better outcomes while minimizing overfitting. However, when limited data are available, a MobileNet-v2, which already has a considerable number of pre-trained parameters that allow for effective learning from a small number of H&E-stained images, is preferred.

## Figures and Tables

**Table 1:** Hyperparameters of the MobileNet-v2 model used for the fibroglandular breast density classification of H&E-stained images.

Hyperparameter	Value
Batch size	32
Maximum allowable number of epochs	200
Patience	10
Learning rate	0.001
Learning rate scheduler	Exponential
Step size of the learning rate scheduler	1
Learning rate decay rate	0.9
Loss function	Categorical cross entropy
Optimiser	Adam

**Table 2:** Details of layers and the number of trainable and all parameters for each architectural model of MobileNet-v2.

MobileNet Architecture	Added GAvgP-2D Layers	Added Dense Layers	Added Dropout Layers	Added BatchNorm Layers	Added Conv2D Layers	Added MaxP2D Layers
MobileNet-Arc 1	1	3	3	2	0	0
Number of trainable parameters: 175,365						
Number of total parameters: 2,436,165						
MobileNet-Arc 2	1	2	2	1	0	0
Number of trainable parameters: 84,869						
Number of total parameters: 2,345,413						
MobileNet-Arc 3	1	2	2	1	1	1
Number of trainable parameters: 741,957						
Number of total parameters: 3,000,069						
MobileNet-Arc 4	1	5	5	3	0	0
Number of trainable parameters: 177,861						
Number of total parameters: 2,438,789						

**Table 3:** Precision, sensitivity, and F1-score of the transferred MobileNet-v2 model with four different architectures of the added layers.

Model Architecture	Breast Density Score (Class)	Precision	Recall (Sensitivity)	F1-Score
MobileNet-Arc 1	1	0.95	0.95	0.95
	2	0.91	0.92	0.92
	3	0.91	0.86	0.89
	4	0.90	0.93	0.92
	5	0.96	0.96	0.96
MobileNet-Arc 2	1	0.96	0.93	0.95
	2	0.89	0.92	0.90
	3	0.88	0.88	0.88
	4	0.92	0.88	0.90
	5	0.94	0.96	0.95
MobileNet-Arc 3	1	0.88	0.91	0.89
	2	0.82	0.80	0.81
	3	0.73	0.84	0.78
	4	0.89	0.73	0.80
	5	0.88	0.90	0.89
MobileNet-Arc 4	1	0.95	0.95	0.95
	2	0.89	0.92	0.91
	3	0.91	0.87	0.89
	4	0.89	0.92	0.91
	5	0.95	0.94	0.95

**Table 4:** Hyperparameters of the ViT model used for the breast density classification of H&E-stained images of human breast.

Hyperparameter	Value
Batch size	64
Maximum number of epochs	75
Patience	15
Learning rate	0.001
Learning rate scheduler	StepLR
Step size of the learning rate scheduler	1
learning rate decay factor (gamma)	0.7
Loss function	Cross Entropy Loss
Optimiser	Adam

**Table 5:** The details of the architecture of the four different ViT models. The total number of trainable parameters was given for each model architecture.

Model	Patch Size	Embedding Dimension	Depth	Heads	MLP Dimension	Dropout	Embedding Dropout
ViT model 1	16	64	12	8	64	0.1	0.1
Number of trainable parameters: 1,740,549							
ViT model 2	32	32	12	8	64	0.1	0.1
Number of trainable parameters: 945,061							
ViT model 3	16	64	6	4	32	0.05	0.05
Number of trainable parameters: 484,293							
ViT model 4	16	64	3	2	16	0.02	0.02
Number of trainable parameters: 169,653							

**Table 6:** Precision, sensitivity, and F1-score of the ViT model with four different architectures.

Model	Breast Density Class	Precision	Recall (Sensitivity)	F1-Score
ViT model 1	1	0.97	0.88	0.92
	2	0.91	0.92	0.91
	3	0.91	0.95	0.93
	4	0.94	0.93	0.93
	5	0.96	0.99	0.97
ViT model 2	1	0.86	0.95	0.91
	2	0.85	0.88	0.86
	3	0.92	0.84	0.87
	4	0.88	0.90	0.89
	5	0.96	0.91	0.93
ViT model 3	1	0.90	0.98	0.94
	2	0.94	0.91	0.92
	3	0.96	0.95	0.95
	4	0.90	0.94	0.92
	5	0.98	0.91	0.95
ViT model 4	1	0.93	0.95	0.94
	2	0.85	0.94	0.90
	3	0.96	0.84	0.90
	4	0.89	0.91	0.90
	5	0.96	0.94	0.95

**Figure A1:**
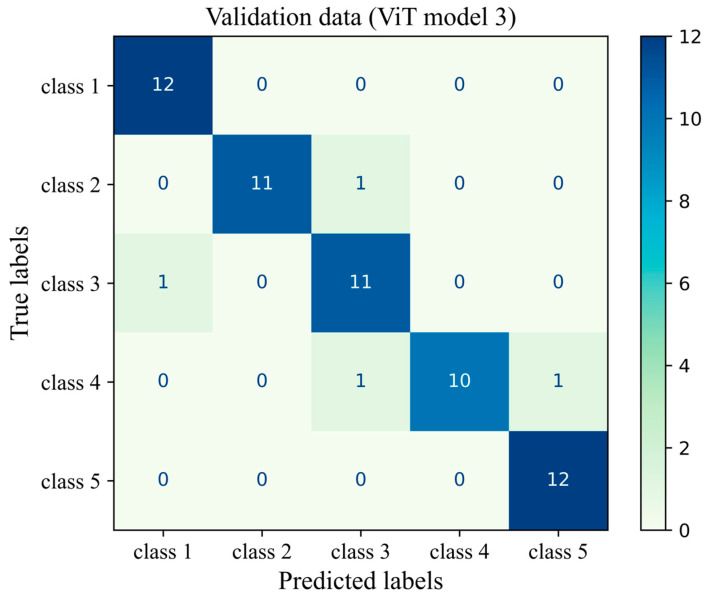
Confusion matrix of the ViT model 3 for the unseen validation H&E-stained images of human breast tissue.

## Data Availability

Data available upon request.

## Appendix A

### Appendix A.1. Validation Study

To verify the results of the present deep learning model, a validation study was performed using H&E-stained images from another laboratory. It is crucial to test the capability of the model and its performance on external data sources generated by different personnel and using different equipment. This minimizes the risk of potential bias toward a specific imaging protocol or sectioning and staining methods. The validation set consisted of tissue sections, which had been prepared and H&E-stained using the same protocol but using different reagents and processing and sectioning equipment and different researchers. Sixty different images were used in the validation study. The fibroglandular density of the H&E-stained images was classified as described in Section 2.3 and was uniformly distributed across all breast density classes (12 images in each class), allowing for a reasonable assessment of the model performance in each class.

The accuracy score, mean precision, average sensitivity, and F1-score were calculated and presented in Table A1. In addition, the precision, sensitivity, and F1-score of the ViT model on each density class were measured. Figure A1 gives more details about the classification by visualizing the confusion matrix of the ViT model 3 for the unseen validation of H&E-stained images of human breast tissue. To minimize the ViT model’s bias on our experimental system and to allow for domain adaptation, stain normalization was conducted using a Reinhard normalizer. This was conducted using one target image from the H&E-stained validation sample set, which showed clear staining of blue nulcei and pink cytoplasm and extracellular matrix. The architectural configuration of the deep learning model was set the same as that of the ViT model 3, which had the best overall performance metrics on the original dataset of H&E-stained images. The accuracy of the deep learning model on unseen validation data was 93%. The mean precision, sensitivity, and F1-score were 94%, 93% and 93%, respectively. These findings supported the capability of the model on the fibroglandular breast density classification of unseen H&E-stained images from another independent source and it demonstrates the flexibility of the model for domain adaptation. However, the model’s precision and F1-score on the target image distribution are, respectively, 9% and 6% lower than those of the original data distribution for class 3, highlighting the need for more domain adaptation processes to improve model performance on unseen data from different sources.

**Table A1:** Validation study: precision, sensitivity, and F1-score of the ViT model tested on H&E images generated in a different laboratory.

Performance Metrics *	Breast Density Class	Precision	Recall (Sensitivity)	F1-Score
	1	0.92	1.0	0.96
	2	1.0	0.92	0.96
	3	0.85	0.92	0.88
	4	1.0	0.83	0.91
	5	0.92	1.0	0.96
Micro-average		0.94	0.93	0.93

* The overall accuracy score on the validation dataset from another laboratory is 0.93.

### Appendix A.2. Ablation Study

In order to study the effectiveness of each module or modeling technology, an ablation study was conducted for both the MobileNet-v2 model and the ViT model (Table A2). The baseline model of the MobileNet-v2 was taken as GAP-2D = 1, DL = 2, DOL = 2, BNL = 1, Conv-2D = 1, and MaxP-2D = 1 where GAP-2D, DL, DOL, BNL, Conv-2D, and MaxP-2D were the global average pooling, dense layers, dropout layers, batch normalization layers, convolutional two-dimensional layer, and the maximum pooling layer, respectively. To investigate the effect of the two-dimensional convolution, the Conv-2D layer was removed. It was found that the removal of this module had a significant increasing effect on the performance metrics of the MobileNet-v2 model, leading to better breast density classification. Furthermore, a small increase in the number of DL, DOL, and BNL (an increase of one unit) slightly improved the performance metrics of the MobileNet model.

To study the effect of patch size and the embedding process (embedding process ablation), we considered two cases: (1) patch size = 16 and embedding dimension = 64, (2) patch size = 32, and embedding dimension = 32. The second case possessed larger patches with a lower embedding dimension. The model of the case study one demonstrated a better performance since it was capable of capturing more details of a given H&E image by dividing the image into smaller pieces and adopting a higher embedding dimension. When a smaller embedding dimension was implemented, less information was encoded and conveyed across the deep-learning modeling pipeline. In another case study, we halved the number of depths, heads, and MLP dimensions of the ViT model to explore the influence of transformer encoders. Although the capacity of the transformer encoder to capture patterns and different features was reduced, the performance of the model did not significantly change, as evidenced by the average F1-score. This is due to the fact that complex transformer encoders were more prone to overfitting. If we further decreased the number of depths, heads, and MLP dimensions of the ViT model, it did not improve the model performance as it reduced the capability of the ViT model to take into account crucial features of H&E images. Therefore, a proper balance and trade-off between the transformer encoder’s complexity and computational resources is required before the widespread application of the deep learning model.

**Table A2:** Ablation study: the effectiveness of each module or component of deep learning models.

Deep Learning Model	Configuration	Precision	Sensitivity	F1-Score
MobileNet-v2	Baseline model	0.840	0.836	0.834
	Baseline model without any Conv2D layers (DL = 2, DOL = 2, BNL = 1)	0.918	0.914	0.916
	Baseline model without any Conv2D layers but with more dense layers (DL = 3, DOL = 3, BNL = 2)	0.926	0.924	0.928
	Baseline model (case study 1)	0.938	0.934	0.932
ViT	ViT model 3 with a weaker embedding process (case study 2)	0.894	0.896	0.892
	ViT model 3 with half capacity of transofermer encoders (case study 3)	0.936	0.938	0.936
	ViT model 3 with quarter capacity of transofermer encoders (case study 4)	0.918	0.916	0.918

### Appendix A.3. Comparison Study

Table A3 lists the details of a number of deep learning models developed for breast density classification, including both mammography images and H&E histopathology images (the present modeling). The type of the model, the number of images used for training, the pre-processing techniques, the data splitting method, and the accuracy are presented. Wu et al. [44] developed a CNN model for the breast density classification of mammography images and trained it on around 200,000 mammograms. Their model achieved an accuracy score of 77% in the four-category breast density classification of mammograms as recommended by the American College of Radiology (the ACR mammographic density classification). Using advanced pre-processing techniques, the Mammographic Image Analysis Society (MIAS) database, and a CNN approach, Shi et al. [45] improved the accuracy of the ACR breast density classification by about 7%. In addition, DenseNet-SE [46], InceptionV3 [47], VGG16 [48], and ResNet-18 [36] were successfully used to classify mammography images based on breast density. In the present work, we have developed two different deep learning models, including a ViT algorithm and a MobileNet-v2 model, to determine the breast density score of H&E-stained human breast tissue sections for the first time. The accuracy scores of the ViT and MobileNet-v2 models were obtained as 94% and 93%, demonstrating the high capacity of these models in the breast density classification of H&E images of human breast tissues.

**Table A3:** Comparison study: the breast density classification for both mammograms and H&E images (the present model).

Image Type	Model	Dataset	Pre-Processing	Data Splitting	Accuracy
Mammogram	CNN [32]	200,000 images	Augmentation	Test: 10%, Training: 80%, Validation: 10%	0.77
Mammogram	CNN [33]	MIAS *	Augmentation, resizing and segmenting	Test: 20%, Training: 80% (five-fold cross validation)	0.84
Mammogram	VGG16 [36]	1602 images	Augmentation	Test: 30%, Training: 70%	0.80
Mammogram	DenseNet-SE [34]	18,157 images	Backgraound removal, grayscale transformation, augmentation, and normalisation into a Gauss distribution	Ten-fold cross validation	0.92
Mammogram	ResNet-18 [37]	41,479 images	Augmentation	Test: 20%, Training: 80%	0.77
Mammogram	InceptionV3 [35]	3813 images	Backgraound removal, and augmentation	----	0.83
H&E	MobileNet-v2 (present work)	965 images	Augmentation and stain normalisation	Test: 25%, Training: 75%	0.93
H&E	ViT (present work)	965 images	Augmentation and stain normalisation	Test: 15%, Training: 85%, Validation: 33% of the test	0.94

* Mammographic image analysis society (MIAS) database.
